# DNAzymes: Expanding the Potential of Nucleic Acid Therapeutics

**DOI:** 10.1089/nat.2022.0066

**Published:** 2023-06-02

**Authors:** Leon M. Larcher, Ianthe L. Pitout, Niall P. Keegan, Rakesh N. Veedu, Sue Fletcher

**Affiliations:** ^1^ Centre for Molecular Medicine and Innovative Therapeutics, Murdoch University, Perth, Australia.; ^2^ Discovery, PYC Therapeutics, Nedlands, Australia.; ^3^ Precision Nucleic Acid Therapeutics, Perron Institute for Neurological and Translational Science, Perth, Australia.

**Keywords:** nucleic acids, DNAzyme, DNA catalyst, delivery, therapeutics molecular medicines, central nervous system

## Abstract

Nucleic acids drugs have been proven in the clinic as a powerful modality to treat inherited and acquired diseases. However, key challenges including drug stability, renal clearance, cellular uptake, and movement across biological barriers (foremost the blood–brain barrier) limit the translation and clinical efficacy of nucleic acid–based therapies, both systemically and in the central nervous system. In this study we provide an overview of an emerging class of nucleic acid therapeutic, called DNAzymes. In particular, we review the use of chemical modifications and carrier molecules for the stabilization and/or delivery of DNAzymes in cell and animal models. Although this review focuses on DNAzymes, the strategies described are broadly applicable to most nucleic acid technologies. This review should serve as a general guide for selecting chemical modifications to improve the therapeutic performance of DNAzymes.

## Introduction

Nucleic acid therapeutics, including antisense oligonucleotides (ASOs), have attracted significant interest in recent years for the treatment of inherited diseases [1]. Several antisense drugs designed to modify mRNA splicing, and thereby restore functional protein expression, were approved from 2016 to 2021 [2]. ASO technologies provide a powerful method for changing gene expression and treating some of the deadliest diseases afflicting humanity. The effectiveness of ASOs has been demonstrated in the clinic with the use of antisense drugs for Duchenne muscular dystrophy, including Eteplirsen (ExonDys51^®^) [3,4], Golodirsen (VyonDys53^®^) [5], and Casimersen (Amondys45^®^) [6], and for spinal muscular atrophy, Nusinersen (Spinraza^®^) [7,8]. Other types of synthetic nucleic acids, including small interfering RNAs (siRNAs) in the clinic since 2018, small activating RNAs, long noncoding RNAs, regulatory RNAs, aptamers, DNAzymes, and micro RNAs and antigomirs, are at various stages of preclinical and clinical development [9–15].

In the 1980s the discovery of ribozymes (catalytic RNA molecules) [16,17] inspired researchers to search for analogous DNA enzymes. The first such “DNAzyme” was made by substituting ribonucleotides with deoxyribonucleotides in the catalytic core of a hammerhead ribozyme sequence isolated from a plant virus [18]. Like the original ribozyme, this DNAzyme was capable of binding and cleaving sequence-specific sites in RNA molecules. All subsequent DNAzymes have been synthetically developed through SELEX (systemic evolution of ligands by exponential enrichment); naturally occurring DNAzymes, although theoretically possible, have not yet been found.

**FIG. 1.**
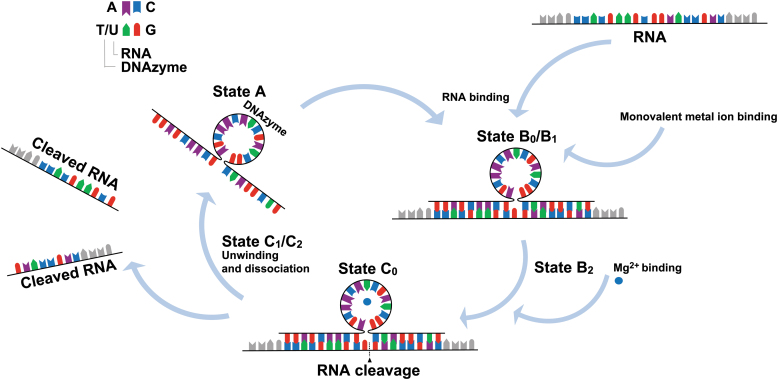
Stages of DNAzyme-mediated RNA cleavage. For simplicity, conformationally similar stages have been elided together (see Borggräfe *et al.* [23] for detailed binding kinetics). The unassociated DNAzyme (state A) binds to the target RNA (state B_0_). Monovalent metal ions bind, stabilizing the DNAzyme/RNA complex (state B_1_). Cofactor (M^2+^) (e.g. Mg^2+^, Ca^2+^, Na^+^, etc.) binding causes conformational activation of the DNAzyme (state B_2_). Once M^2+^ equilibrium is reached the substrate is cleaved (state C_0_). M^2+^ is released and the DNAzyme unwinds and dissociates from the cleaved RNA (state C_1_/C_2_). This process may repeat over multiple cycles. Adapted from Ref. Chakravarthy *et al.* [1].

**FIG. 2.**
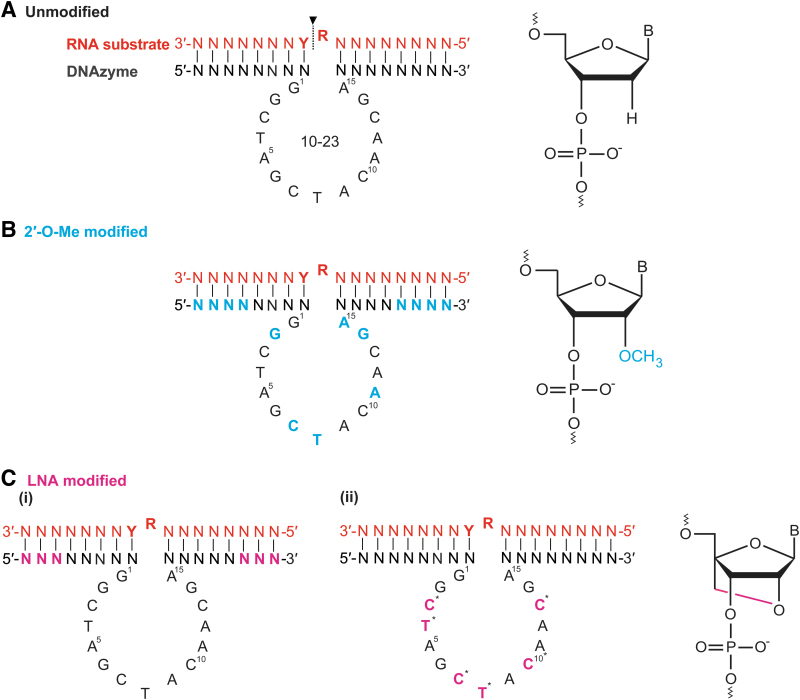
Modified and unmodified 10–23 DNAzymes. A/C/G/T = specific nucleotides, R = purine, Y = pyrimidine, N = any nucleotide, B = any nucleotide base, P = phosphate, O = oxygen and C = carbon. Nucleotide color denotes chemistry: *red* = RNA, *black* = DNA, *blue* = 2′-*O*-Me, and *pink* = LNA. **(A)** Unmodified DNAzyme. The DNAzyme binds an RNA substrate and cleaves it primarily between a purine (R) and a pyrimidine (Y) (cleavage site indicated by *arrow*). **(B)** A widely used 2′-*O*-Me modification scheme, with four or five 2′-*O*-Me modifications at the 3′ and 5′ ends and six modifications in the core at positions G2, C7, T8, A11, G14, A15. **(C)** Common LNA modifications are at either **(i)** the first and last three nucleotides of the arms, or **(ii)** individual nucleotides of the core. As denoted by the *asterisks*, only one of these indicated nucleotides is LNA chemistry in any DNAzyme of this design. Adapted from Ref. Santoro and Joyce [21]. 2′-*O*-Me, 2′-*O*-methyl; LNA, locked nucleic acid.

In 1997, improved SELEX criteria led to the creation of what are now the most widely used DNAzyme: The 10–23 DNAzyme, so named for being isolated from clone 23 after 10 rounds of SELEX amplification [19]. The 10–23 DNAzyme structure consists of two flanking arms, which bind a specific RNA target through Watson–Crick base pairing, and a 15-nucleotide inner catalytic loop that cleaves phosphodiester (PO) bonds (primarily purine:pyrimidine) in the center of the mRNA binding site [20] (Figs. 1 and 2). Owing to the need to balance target specificity against molecule size, 10–23 DNAzymes are typically no shorter than 25 nucleotides and no longer than 37 nucleotides in length.

Santoro and Joyce, Ponce-Salvatierra *et al.*, and Borggräfe *et al.* have explored in detail the specific folding or conformational changes that occur as DNAzymes bind to mRNA and exert catalytic function [21–23]. In brief, as outlined in Fig. 1, the following folding states (B_0–_C_0_) are essential for DNAzyme mode of action: (1) binding arm recognition and annealing to the specific target sequence transitions the DNAzyme to state B_0_, forming a heterogeneous precatalytic DNAzyme/RNA complex, (2) monovalent metal ion binding stabilizes the complex, causing conformational activation without promoting cleavage (state B_0_–B_1_), (3) upon binding of M^2+^, B_1_ transitions to a catalytically active complex state B_2_, (4) once M^2+^ equilibrium is reached, the DNAzyme transitions to state C_0_ in which substrate cleavage occurs, (5) following cleavage, M^2+^ is released, the DNAzyme core unwinds but remains bound to the cleaved substrate, entering either state C_1_ or C_2_ both of which have a destabilized core region and likely undergo a slow conformational exchange process.

State C_1_ is predominant at 37°C, whereas state C_2_ occurs at higher temperatures. The transition into states C_1_ and C_2_ may not always occur, instead remaining in state C_0_ (catalytically active structure) bound to the RNA substrate (cleaved product remains bound to an activated catalytic core). Finally, the cleaved product dissociates from the DNAzyme [23]. These unusual structural properties of DNAzymes ensure highly specific RNA cleavage at targeted junctions, in comparison with other antisense technologies, including RNase H competent ASOs or siRNA [20]. In addition, DNAzymes possess the ability to cleave all types of RNA molecules (messenger RNA, pre-microRNA, and microRNA, etc.) at almost any position.

As potential therapeutic agents, DNAzyme technology offers expedient characteristics, such as lower synthesis costs, self-catalytic ability allowing RNase H independence, and notably, the cleavage of the target RNA is repeated over several cycles of annealing (state B_0_) and cleavage (state C_0_) (multiple turnover) (Fig. 1) that may allow low DNAzyme dosages for sustained therapeutic effect [24].

Although DNAzymes show promise, they face several challenges to successful therapeutic application including: limited half-life and intracellular stability, poor delivery and retention in target tissues, off-target binding to RNA and proteins, inefficient cellular penetration, and limited endosomal escape. Another obstacle to DNAzyme therapeutics is the requirement for high concentrations of endogenous cofactors (Mg^2^, Ca^2+^, Na^+^, etc.), to maintain catalytic activity in living cells. However, recent data from Borggräfe *et al.* suggests that neither Mg^2+^ turnover or reaching the Mg^2+^ equilibrium are rate-limiting steps of DNAzyme catalysis, but rather influence transition from state B_2_ to C_0_ [23].

Because phosphate bonds are cleaved by exonucleases and the nucleotides are removed by glomerular filtration, natural nucleic acids are extremely vulnerable to destruction by plasma enzymes and bodily fluids [25]. For macromolecules, the renal filtration limit is between 2 and 4 nm. Hence, systemically administered oligonucleotides are removed more quickly and do not reach the renal filtration threshold. In addition, the half-life of unmodified nucleic acids varies from a few minutes to a few hours [26–28], resulting in insufficient levels for therapeutic impact and decreased accumulation near the target [29]. Therapeutic nucleic acids must be stable in the plasma, penetrate the targeted tissue, and remain there for an extended period to be effective. In addition, once within the cell, the nucleic acid must escape the endosomal compartment to circumvent degradation by RNases and engage with target RNA [29,30]. Owing to the negative charge and large size of nucleic acids [∼10–17 kDa for an ASO (10–25 bp) and 14 kDa for double-stranded siRNA (20 bp)], and their consequently inefficient transiting of cell membranes, local ASO administration only produces modest intracellular delivery.

Nucleic acid drug delivery remains an area of intense interest and lies on the critical path in drug development. Both local and systemic injection of nucleic acid drugs to treat a range of conditions, including neurodegenerative diseases have been explored, with variable outcomes. Major improvements in nucleic acid drug delivery and efficacy in target cells, in the central nervous system (CNS) in particular, will be required to realize the potential of these drugs.

Neurodegenerative diseases are maladies predominantly affecting the structure or function of the brain and spinal cord [31]. Conditions such as amyotrophic lateral sclerosis, Parkinson's disease, frontotemporal dementia, Alzheimer's disease, and multiple sclerosis, can be attributed to both genetic and environmental factors [32]. Despite the debilitating nature, progressive degeneration, fatal outcomes, growing impact of disease and rapidly increasing prevalence, there is a clear lack of effective treatments and no cure for most neurological disorders. Current treatments for neurodegenerative diseases are only moderately effective in managing symptoms (symptomatic and supportive treatments) and do not prolong life, nor improve quality of life significantly [33,34]. Even marginal improvements in treatment outcomes could have substantial benefits and help reduce some of the impacts caused by this group of diseases. The development of nucleic acid therapeutics and effective delivery methods has the potential to address this unmet need.

The effective utilization of nucleic acid therapies for the treatment neurological disorders requires drug transport to the CNS (brain and spinal cord). However, the tightly regulated barriers (physical and physiological) that safeguard the brain from infectious and poisonous substances, namely the blood–brain barrier/cerebral endothelium [35], present particular challenges to systemically administered therapeutics. Three main modifications are used to improve stability and delivery of DNAzymes; chemical modification incorporated directly into the DNAzyme molecule and encapsulation of the DNAzyme or conjugation of a functional domain.

## Chemical and Structural Modifications

Modification of the DNAzyme catalytic core can increase its resistance to endonucleases [36–38], whereas modifications made to the core or arm regions of the DNAzyme can increase stability and catalytic ability. In addition, conjugation of functional groups or transport molecules can also increase the stability and cellular uptake of DNAzymes. In this section, modifications increasing efficacy or stability are discussed.

DNAzymes are susceptible to nucleolytic degradation in biological fluids, limiting their period of action. To extend the *in vivo* half-life of these molecules, modified nucleotides can be incorporated to enhance biostability while maintaining low toxicity, high target affinity [39], and catalytic function. Table 1 summarizes the advantages and disadvantages of each modification.

**Table 1.** Summary of DNAzyme Chemical Modifications, Indicating Advantages and Disadvantages of Each

Chemical modification	Advantage	Disadvantage	References
3′-inverted dT	Improved stability Does not disrupt catalytic function Can improve cleavage	Relatively short half-life	[1,40–43]
Phosphorothioate (PS)	Improved stability Increases cellular uptake	Decrease substrate affinity Toxic side effects	[1,36,42,44–49]
2′-*O*-methyl (2′-*O*-Me)	Improved stability Can be incorporated into core	Reduced cleavage ability	[37,38]
Locked nucleic acid (LNA)	Improved base pairing selectivity and efficiency Reduced sequence length Improved stability Improves binding to highly structured RNAs	Inflexible/rigid structure Reduced cleavage ability Reduced multiple turnover	[1,37,50–52]
2′-***O***-(*N*-(aminoethyl)carbamoyl) methyl	Improved catalytic function		[53–56]
2′-deoxyadenosine analogues	Improved catalytic function		[57]
2′deoxyuridine derivative containing a guanidinium group	Reduced negative charge (increased cellular uptake)	May reduce catalytic ability	[58]
Phosphorodiamidate morpholino oligonucleotides (PMO)	Improved stability Excellent safety profile	Has not been applied to DNAzymes May lower catalytic ability Reduced binding affinity	[59]
2′-fluoroarabino nucleic acid (FANA) and α-l-threofuranosyl nucleic acid backbone (XNA)	Improved catalytic function Improved stability Allele specificity		[60,61]

## Inverted Thymidine

Owing to the single-stranded nature of DNAzymes, under cellular conditions they are promptly degraded by nucleases, including endonucleases and the 3′ exonucleases [37]. Incorporating an inverted thymidine (dT) modification at the 3′ end of a DNAzyme [1,40–43] can increase its intracellular stability by reducing susceptibility to 3′ exonuclease degradation. In many cases, this inverted dT modification also improves the DNAzymes catalytic activity [1]. Recently, Wang *et al.*, used an α-l-threofuranosyl nucleic acid (TNA) thymidine (tT) to cap both the 5′ and 3′ ends of the DNAzyme (X10–23). This modification is almost completely resistant to enzymatic degradation. The authors demonstrated enhanced biostability, with almost no degradation of the modified DNAzyme compared with those without tT modification. Furthermore, these X10–23 tT-modified DNAzymes showed increased catalytic activity: Cleavage was ∼50-fold faster under multiple turnover conditions (higher concentration of DNAzyme in relation to RNA substrate) and ∼3-fold faster under steady-state conditions (concentration of DNAzyme is equal to RNA substrate) compared with the unmodified DNAzyme.

## Phosphorothioate

Despite the extensive use of the phosphorothioate (PS) backbone for the stabilization of oligonucleotides, this modification is known to decrease DNAzyme substrate affinity and thus the efficiency of substrate cleavage (tenfold lower catalytic activity relative to unmodified analogues). The PS modifications are also known to exert toxic off-target effects [1,44–48]. However, the addition of PS modifications to DNAzymes can increase both their resistance to degradation and their cellular uptake [36,42,49]. Chakravarthy *et al.* demonstrated that the addition of PS linkages to the arm regions of their lead DNAzyme increased its nuclease resistance. However, the modification reduced the catalytic ability of the DNAzyme from 29% substrate cleavage to 10% cleavage in human fibroblasts.

Despite the decrease in catalytic ability, resistance to degradation and better uptake of the DNAzyme contributed to the inhibition of gene expression in cultured cells and *in vivo*; in contrast, the unmodified DNAzymes proved ineffective [1]. The PS linkage modifications can be used with base modifications [such as 2′-*O*-methyl (2′-*O*-Me), locked nucleic acid (LNA), etc.); however, toxicity associated with the PS modification may limit the therapeutic utility of PS-modified oligonucleotides in neurological diseases and in other sensitive tissues; because neurons and other postmitotic cells cannot regenerate, any damage caused to the tissue is permanent. It should be noted that efforts to optimize the use of PS modifications in ASOs have been made, the elimination of nonessential PS modifications have allowed retention of the improved ASO stability while contributing toward the minimization of off-target toxicity [62–64].

## 2′-*O*-Methyl Modifications

The widely used 2′-*O*-methylribonucleotide modification, whereby an *O*-methyl group replaces the 2′ hydroxyl of the ribose moiety, can protect a DNAzyme from nuclease degradation [38,65]. Incorporation of 2′-*O-*Me modifications (on a PO backbone) into the DNAzymes catalytic core residues improves its activity and endonuclease resistance; the most commonly used pattern of modifications are applied to the last four to five nucleotides of the DNAzyme arms and to six nucleotides within the core (positions G2, C7, T8, A11, G14, A15) [37,38,60,65], which we will refer to as “a4c6” (Fig. 2B). Single-base modifications in the core showed enhanced cleavage *in vitro* at residues G2, C7, T8, A11, G14, A15 compared with the unmodified DNAzyme [37].

Wiktorska *et al.* showed that in a comparison between a β1-integrin targeting DNAzyme (containing a 2′-*O*-Me substitution in the catalytic core) and siRNA, the modified DNAzyme showed enhanced activity, resulting in greater β1-integrin knockdown in mice. In addition, the modified DNAzyme inhibited cell growth and diminished tumor formation in cell lines (PC3 and HT29 colon cancer) and in a mouse xenograft model, respectively. This suggested that owing to the improved resistance to degradation, the efficacy of the DNAzyme was comparable with siRNA activity in long-term experiments *in vivo* [66].

## Locked Nucleic Acid

LNA is a ribonucleotide modified with a methylene bridge connecting the 2′-oxygen of the ribose with the 4′-carbon locking the ribose moiety in a C3′-endo conformation [67–69]. A hallmark of LNA-modified sequences is extraordinarily high binding affinity and specificity toward complementary RNA or DNA sequences [50,51], and these molecules also show improved base pairing selectivity compared with unmodified nucleic acids, permitting reduced sequence lengths in LNA ASOs.

Several groups have incorporated LNA bases into the arm regions of DNAzymes (or LNAzymes) on a PO backbone, demonstrating increased stability while maintaining efficient or enhanced RNA cleavage *in vitro* and *in vivo* [36,67,70–73]. Vester *et al.* found that LNAzymes markedly enhanced the cleavage of highly organized RNA compared with the unmodified DNAzyme (the binding arms incorporated two LNA nucleotides) [67,70]. Schubert *et al.* designed LNAzymes bearing three or four LNA nucleotides at the 3′ and 5′ ends of their binding arms, demonstrating high cleavage efficiency but low efficiency over multiple cleavage cycles (multiple turnover), indicating high target RNA binding affinity [37].

Schubert *et al.* later showed that these same LNAzymes (incorporating three to four LNA monomers) could improve cleavage of unfavorable target structures (tertiary structures that could prevent binding) through improving target affinity [52]. The improved target affinity and base pairing selectivity afforded by LNA modifications are especially advantageous properties for allele discrimination, targeting SNPs or other small mutational differences, or when decreased arm length is essential. More recently, Wang *et al.* further demonstrated the use of the three-LNA modification scheme [Fig. 2C (i)], showing an increase in activity compared with unmodified DNAzyme under both single- and multi-turnover conditions. However, this increase in activity was much lower than that conferred by 2′-fluoroarabino nucleic acid (FANA) modifications of the same DNAzyme sequence [60]. Despite the improvement in target affinity for highly structured RNAs, the incorporation of LNAs should be approached with caution. Excessive affinity for the target RNA molecule can reduce multiple turnover and diminish the efficacy of the DNAzyme, as the DNAzyme product complex may be unable to dissociate from the postcatalytic state [60].

Evidence suggests that LNA incorporation can reduce DNAzyme cleavage activity. Chakravarthy *et al.* demonstrated that incorporating two LNA nucleotides into the arms of their DNAzyme resulted in decreased cleavage of the target *ITGA4* when tested in normal human fibroblasts. Both PS-modified DNAzymes and LNAzymes showed drastically reduced cleavage efficiency [1]. This may be owing to (1) target inhibition due to the cleaved product remaining bound to the LNAzyme, and (2) asymmetrical addition of LNA residues resulting in misalignment of DNAzyme with the target site. LNAs incorporated into certain positions may be incompatible with DNAzymes because of their reliance on specific tertiary structure formation to function.

Modification to the core of the DNAzyme with an LNA may also prevent proper tertiary structure formation of the catalytic core and could drastically reduce catalytic activity. Robaldo *et al.* showed that a single LNA incorporated at individual cytosines (C) within the core at positions C3, C7, C10, C13 [38] and LNA modification to the thymidine (T) residues at positions T4 and T8 individually and in combination [74] (Fig. 2C) almost completely diminished catalytic activity *in vitro*. Although product inhibition would not occur by modifications in the core region, diminished activity is either owing to disruption of the specific folding required for DNAzyme function or generation of unsuitable conformations, in essence “locking” a DNAzyme into an inflexible structure. Plasticity of T4 is particularly important to promote a conformation switch, allowing the DNAzyme-target stabilized complex (state B_1_) [23]. However, some core residues modified with LNAs may tolerate or even benefit from a more conformationally constrained modification.

Recent insights from Borggräfe *et al.* indicate that positions G2 or G14 may act to anchor the DNAzyme to the target, thereby promoting DNAzyme stabilization; transition into state B_1_ (stabilized inactive complex), and possibly anchoring, may be enhanced by the incorporation of LNAs that diminish the rate of product inhibition owing to the lack of direct involvement of G2 and G14 in target binding. Evidence suggests that modification of G2 with 2′-*O*-Me, or FANA and 6-thio at G14 can increase the activity of the DNAzyme [23,37,60], indicating that G2 and G14 may benefit from increased rigidity and/or binding affinity, helping to counteract electrostatic repulsion of the phosphate backbones.

This evidence suggests that LNA modifications in certain positions are a viable strategy for increasing DNAzyme stability and target affinity; however, the incorporation of LNA nucleotides should be carried out systematically. Careful consideration should be given to the binding arm length, sequence arrangement and content, and number of LNA nucleotides/monomers to maintain the DNAzyme catalytic ability and multiple turnover. In addition, the increased binding affinity of LNA nucleotides could promote off-target binding to mismatched and shorter RNA substrates.

## Additional Chemical Modifications

Several nucleotides in the DNAzymes catalytic core have been replaced by alternative natural or synthetic nucleotides [75]. The inclusion of functional groups, as opposed to nucleobase replacement resulted in more effective cleavage activity when specific nucleobases in the DNAzymes catalytic core were modified [37]. Other 2′-substitutions such as the addition of positively charged end amino groups, such as 2′-*O*-carbamoylmethyl, 2′-*O*-(*N*-(aminoethyl)carbamoyl)methyl [55,56], and 2′-*O*-(3-amino)propyl groups [53], can improve delivery (uptake) and stability (nuclease resistance) of the nucleic acids [76].

To introduce different functional groups into the 6-amino position and analyze their contribution to catalytic activity, Zhu *et al.* incorporated several 2′-deoxyadenosine analogues into the five adenine (A) residues of the core region. A five and twofold increase in catalytic activity resulted from these 3-aminopropyl substitutions on the 6-amino group positions at A9 and A15, respectively. The 6-amino group of all adenines, with the exception of A15, influenced activity either through folding or cleavage; the unmodified 6-amino group of A15 appeared to be detrimental to the cleavage activity [57], indicating room for further optimization of the catalytic core.

Du *et al.* introduced a 2′*-O*-(*N*-(aminoethyl)carbamoyl) methyl group into the adenosine residues of the catalytic core at the 2′ position. They demonstrated that the 2′-*O*-(*N*-(aminoethyl)carbamoyl) methyl effectively increased catalytic activity (*in vitro*) when placed within particular regions of the DNAzyme (A15), and hypothesized that, owing to its ability to form hydrogen bonds, the modification was able to induce favorable conformational interactions [54].

Lam and Perrin designed a DNAzyme with a 2′deoxyuridine derivative modified with a guanidinium group integrated into the binding arm. The modification did not lead to improvement in catalytic ability; however, the 10–23 DNAzyme had reduced negative charge that could potentially enhance cellular uptake. It should be noted that this hypothesis is yet to be confirmed in cells [58].

Recently Wang *et al.* designed XNAzymes. The 10–23 DNAzymes were modified by incorporation of different molecular chemotypes, FANA [61] was used in both the binding arms and at catalytic core positions G2 and U8, and TNA backbone architecture [77] was used to cap both the 5′ and 3′ ends of the XNAzyme (X10–23). The authors reported increased biological stability and enhanced mRNA cleavage [60]. Taking this further, these researchers designed another DNAzyme using the same optimized X10–23 design (XNAzyme). The DNAzyme targeted a SNP (G12V) in the Kristen rat sarcoma virus (*KRAS*) gene associated with disease. The DNAzyme cleaves the G12V RNA substrate specifically at a G-U junction with >80% substrate cleavage in 1 h, with no RNase H activity *in vitro* targeting template RNA. G12V mRNA was reduced by >50% following administration of 10–23 into NCI-H441 cancer cells, resulting in prominent decreases in KRAS protein expression [78]. Overall, Wang *et al.* and Nguyen *et al.* showed that the XNAzyme modification can increase DNAzyme stability, specificity, and cleavage efficiency compared with other chemical modifications and unmodified DNAzymes [60,78].

Additional modifications that have proven effective in ASOs could be used in DNAzymes, such as 2′-MOE and phosphorodiamidate morpholino oligonucleotides (PMOs). The 2′-MOE is a 2′ ribose substitution similar to 2′-*O*-Me and 2′-fluoro, whereas PMOs substitute neutral phosphoryl guanine groups for anionic PO groups [59]. Both these modifications can potentially improve the uptake, stability, and efficacy of DNAzymes *in vivo*. However, to date, the impact of these additional modifications to DNAzymes is yet to be explored.

A potential drawback of the PMO chemistry for use in DNAzymes is the synthetic nature (large structural differences of PMO vs. DNA); a mixed chemistry can be difficult to achieve owing to difficulty in chemical synthesis (likely a ligation method would need to be used to “stick” together segments of PMO and DNA, potentially dramatically increasing cost, limiting synthesis yield, and purity). In addition, full PMO modification may present similar challenges as other fully or heavily modified 10–23 DNAzymes, that is, the significant loss of cleavage efficiency. It should be noted that the recently used hemin/g-quadruplex DNAzyme modified with PMO outperformed its DNAzyme analogue [79], although folding structure and functional mechanisms differ between the 10–23 DNAzyme and the hemin/g-quadruplex DNAzyme; this study indicates potential utility of PMO modifications to 10–23 DNAzymes.

Thiomorpholinos oligonucleotide chemistry [80,81] may offer a way to sustain or improve catalytic ability and potentially mitigate some of the toxicity associated with 2′-*O-*Me and PS linkages. The hybrid structure may provide both improved stability and binding affinity while allowing routine and more straightforward mixed chemistry synthesis, and also allowing transferability of modifications that have been developed for other, similar chemistries, that is, 2′-*O-*Me, with minimal “tweaking” for individual DNAzymes.

## Noncarrier Molecule Modifications

DNAzyme conjugation to other molecules is a potential strategy to improve stability and nuclease resistance while at the same time improving delivery and cellular uptake. Some conjugations include aptamers [82], hairpin DNAzymes (hpDNAzyme), “caged” nucleic acids, and nucleic acids displaying nanostructure acids. In this section, only molecules that primarily increase DNAzyme intracellular stability are discussed (noncarrier molecules), whereas molecules that primarily increase cellular uptake and delivery (carrier molecules) such as aptamers and peptides are not discussed in this review. The advantages and disadvantages of each system are summarized in Table 2.

**Table 2.** Summary of Advantages and Disadvantages of DNAzyme Noncarrier Molecules

Noncarrier molecules	Advantage	Disadvantage	References
Hairpin modification/leash sequence	Improves stability Low/toxicity/immunogenicity Does not disrupt catalytic function	Relatively short half-life	[83,84]
“Caged” DNAzyme	Does not disrupt catalytic function On/off control Improved stability	Toxicity/immunogenicity is unknown	[85–91]

### Hairpin DNAzyme

**FIG. 3.**
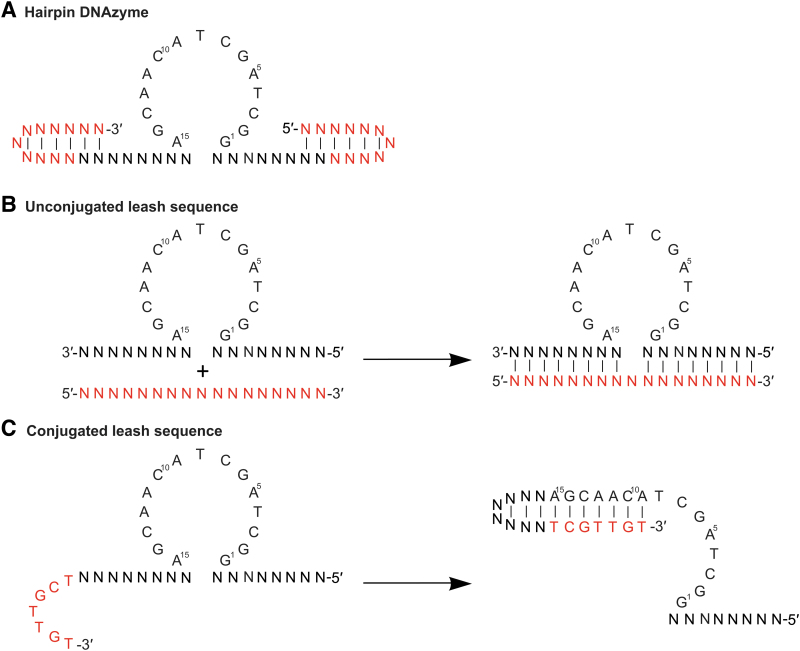
Common noncarrier molecules and moieties utilized with DNAzymes. A/C/G/T = specific nucleotides, N = any nucleotide, *Red* = additional nucleotides added to base DNAzyme sequence. **(A)** hpDNAzyme. **(B)** Unconjugated leash sequence. **(C)** Conjugated leash sequence/overhang, the sequence given is an example and may vary depending on the design. hpDNAzyme, hairpin DNAzymes.

hpDNAzymes are DNAzyme sequences that incorporate stem-loop hairpins at either end of the DNAzyme binding arms (3′ and 5′) [84] (Fig. 3). The addition of stem loops to the DNAzyme may subvert/minimize the need for chemical modification made directly into the DNAzyme molecule, potentially having little to no effect on the catalytic ability. Abdelgany *et al.* designed a unique 10–23 DNAzyme (hpDNAzyme) structure incorporating stem-loop hairpins at the ends of the DNAzyme arms (3′ and 5′). The hpDNAzymes showed exceptional resistance to nucleolytic degradation and demonstrated that hpDNAzymes had a higher efficacy than unmodified DNAzymes at cleaving target mRNA in cells [83].

### Leash sequence (DNA/RNA, DNA/DNA duplexed oligonucleotides)

Leash sequences are DNA sequences (complementary to the arm and/or catalytic regions or a poly G tract) that are conjugated or cotransfected (duplexed) with the DNAzyme (Fig. 3). Binding of the leash sequence to the DNAzyme resulted in the formation of a duplex (made up of double-stranded DNA or RNA) and allowed the “escort” of the DNAzyme to target tissues [84,92–94]. In addition, DNAzyme stability is increased owing to the DNA/RNA or DNA/DNA duplex region being inherently more stable than single-stranded (ssDNA) DNA (DNAzyme) in the cellular environment. Under specific conditions (change in pH, UV radiation, presence of a high affinity binding target, or activation of RNase H activity, etc.), the leash sequence will detach from the DNAzyme, allowing target binding and cleavage. Once detached from the DNAzyme, the leash sequence is usually degraded by cellular machinery.

Two DNAzyme constructs containing 10 guanine residues at the 3′ end and two 12 bp stem-loop hairpin structures on the 3′ and 5′ binding arms were designed by Unwalla and Banerjea [84]. Inclusion of 10 guanine residues incorporated at the 3′ end of the DNAzyme only marginally reduced the cleavage efficiency, showing inhibition of HIV-1 gene expression. In addition, the poly G tract leash/DNAzyme duplex showed marked cellular uptake by macrophage-specific cell lines, without the use of lipofection transfection reagents. Similar strategies expanded on this concept through the incorporation of photoreactive groups for reversible DNAzyme activation (see section Leash sequence (DNA/RNA, DNA/DNA duplexed oligonucleotides)) [84].

The hpDNAzyme and leash DNAzyme molecules are derived from natural DNA or RNA strands; once incorporated these form DNA/RNA heteroduplexes or DNA/DNA homoduplexes, thereby masking sites that are readily degraded, and endowing the molecules with increased uptake and stability to nucleases, without the need for potentially toxic chemical modifications. However, leash sequences can be chemically modified, which confers increased stability, binding affinity, or other favorable qualities. In addition, leash sequences can also be complexed to “carrier” or lipid [95] molecules, endowing the oligonucleotides complexed to the leash with increased organ and cell uptake and penetration. Nagata *et al.* designed a RNase H-dependent ASO (gapmer) that targets metastasis-associated lung adenocarcinoma 1 (*MALAT1*) noncoding RNA. This gapmer was duplexed to a complimentary 2′-*O*-Me–modified RNA strand, with cholesterol or α-tocopherol conjugated to the 5′ end (PS backbone used in the three flanking base the 3′ and 5′ ends).

The authors demonstrated that following subcutaneous or intravenous administration, the DNA/RNA duplex ASO was able to penetrate the CNS in mice and rats. The duplex ASO was distributed throughout the CNS, including the brain and spinal cord, and peripheral tissues with up to 90% suppression of the four genes studied. However, the single-stranded gapmer ASO conjugated to cholesterol showed limited activity in the same model. *MALAT1* was reduced in most CNS cell types, with preferential knockdown in neurons and microglia [93]. Cholesterol-conjugated duplex ASOs may overcome a portion of the efficiency limitations of ASOs targeted to the CNS, without the necessity for invasive intrathecal administration.

For the most part, leash sequences are nontoxic, nonimmunogenic, and biodegradable, thus preventing accumulation and aggregation and showing suitability for use in neurological disorders, especially in nonregenerative cells (eg, neurons). Owing to the duplex formation of the leash and oligonucleotides, off-target binding of endogenous RNA and proteins to the backbone of some chemically modified oligonucleotides [63,94,96] may be mitigated, allowing reduced toxicity of these therapeutic molecules.

### Light-activated “caged” DNAzymes/photocaging

**FIG. 4.**
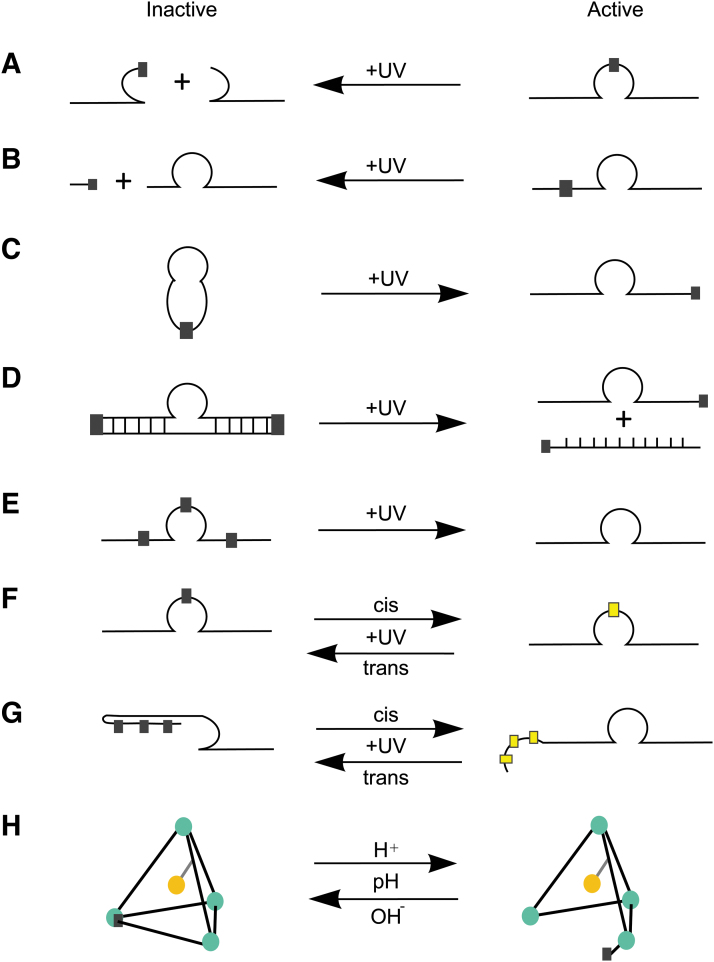
Schematic of light-activated “caged” DNAzymes. **(A)** and **(B)** The photocleavable group (PS/thymidine, indicated by a *square*) in the core region and binding arms causes the DNAzyme to separate upon UV irradiation. **(C**, **D)** Circularized DNAzymes have diminished activity, photolysis of the photocleavable group restores the active form. **(E)** Incorporation of the photocleavable group into catalytic core and arm regions render DNAzyme inactive until removal of the group by UV irradiation activates the DNAzyme. **(F**, **G)** Incorporation of azobenzene nucleotide and overhang complementary to the catalytic core region are reversibly (*cis*-activation/*trans*-deactivation) photo-isomerized by UV irradiation. Adapted from Ref. Richards *et al*.[89]. **(H)** pH-responsive DNA tetrahedral nanocage, the enzyme/DNAzyme is integrated into the tetrahedral structure. Changes to pH allow reversible activation/deactivation.

Similar to the addition of hairpin structures to the DNAzymes, the addition of a photo-protecting group termed “caging” could be used. Light-activated “caged” oligonucleotides or DNA are composed of a photoactivatable linker joining a target-specific strand (ie, DNAzyme, ASO, DNA) to a complementary strand (Fig. 4D, G), itself (Fig. 4C), or at a critical functional motif (Fig. 4A, B, E, F) rendering the molecule inactive. Photocaging can also be applied to nanostructures (Fig. 4G). Young *et al.* showed that introducing a caging group into nucleic acid agents allowed gene silencing in mammalian cells through photochemical regulation [85,86]. This strategy therefore could be extended to DNAzymes.

Application of this strategy to DNAzymes can be used to improve nuclease stability. However, caging of DNAzyme molecules is not restricted to covalently bound linkers—DNA nanocages can also be used to encapsulate the DNAzyme molecule [97] (Fig. 4H). Several caging groups can be included in the DNAzyme molecule, either into the backbone of a DNA oligonucleotide or by direct addition of a caging group on the nucleoside base. Some of these caging groups include azobenzene, phosphonamidite, and TEEP-OH.

Lusic *et al.* developed 10–23 DNAzymes using phosphoramidite chemistry to incorporate light reactive nucleotides at critical positions in the DNAzyme, preventing DNAzyme activity before exposure to UV light [87] (Fig. 4A–E). Reversible DNAzymes were constructed by replacing critical bases using nucleotides modified with a photoisomerizable azobenzene group [88] (Fig. 4F). The 10–23 DNAzymes containing c-azobenzene exhibited higher catalytic activity, and while these strategies allow for photoreversibility, synthesis of azobenzene-modified nucleotides is a requirement.

Richards *et al*. [85–89] designed several photocleavable 10–23 DNAzyme structures targeting *VEGFR2* receptor mRNA using commercially available phosphoramidite chemistry (Fig. 4A–E). The first structure incorporated two photocleavable spacers in the catalytic core and one of the binding arms that rendered the DNAzyme inactive when exposed to UV irradiation (Fig. 4A). The second structure consisted of a circularized DNAzyme, assembled by attachment to a self-complementary strand linked by two photocleavable spacers, remaining inactive pending reactivation by UV light. This strategy eliminated the need for azobenzene nucleotides [89].

Kamiya *et al.* also used a photoresponsive DNA consisting of an overhang that is complementary to the catalytic core regions that comprised of either azobenzene compounds or 2,6-dimethyl-4-(methylthio) azobenzene units, connected to a d-threoinol linker, allowing light-dependent photoswitching DNAzyme activity [98] (Fig. 4G). In these studies, the testing of these caged DNAzymes in cellular systems or the effects of “caging” on nuclease stability was not assessed. However, previous studies showed that the construction of circular DNAzymes by enzymatic ligation [99] or cloning into an expression vector [100] improved the nuclease resistance and cellular delivery of the DNAzymes. Takahashi *et al.* showed that DNAzyme nuclease resistance was increased significantly with the addition of two N3′-P5′ phosphoramidite linkages at the 3′ and 5′ ends, although catalytic activity was retained [101].

DNAzymes containing addition of caging groups on thymidine residues in the catalytic core or the binding arms were constructed. These molecules included caged, trans-acting DNA decoys (complementary sequence) and caged hairpin designs allowing the *cis*-regulation of DNAzyme cleavage activity, demonstrating the light stimulated deactivation of DNAzymes [85] (Fig. 4A–C, E).

Wang *et al.* developed photocaged DNAzymes by the inclusion of a photolabile group (TEEP-OH) into PS DNA. Photolysis of TEEP-OH resulted in the formation of native DNA PO upon light irradiation (Fig. 4E). TEEP-OH modifications within the DNAzyme catalytic core were successfully utilized to cage both 8–17 and 10–23 DNAzymes. DNAzyme designs incorporating three TEEP-OH modifications demonstrated significant off/on activation after light irradiation. Authors also demonstrated intracellular compatibility of the DNAzymes caged with TEEP-OH, suggesting utility for *in vivo* applications [90]. In addition to light-activated caging groups, DNAzyme caging could be achieved by the use of pH responsive acetyl bonds [102,103] or disulfide bridges [104] with the ability to be degraded in the endosomal environment or in the cytosol, respectively.

Xu *et al.* developed a wavelength selective photocaged 8–17 DNAzyme based (Zn^2+^ dependent DNAzyme) (17Dz), containing three PS modifications at the catalytic core. The 17Dz was modified to form two photocaged DNAzymes, a UV-activated DNAzyme modified with 2-(2-nitrobenzyl)oxyphenyl (NBIO-17Dz) and a visible light-activated DNAzyme modified with 7-diethylaminocou-marin (DEACM-17Dz). The DNAzyme cleavage activity was activated *in vitro* and in human cells upon irradiation and exhibited wave-length selective activation in live cells [91].

For the application of caged DNAzyme molecules in neurological disorders, apart from increasing the stability of these molecules, the control of catalytic activity by either external stimulus (light) (ie, probes to be implanted into the brain or near infrared light that can pass through bone and tissue) or owing to changes in cellular environment allow tissue-specific therapeutic activation that in theory may prevent potential off-target effects and saturation of DNAzymes by undesirable targets, potentially allowing the use of lower concentrations of DNAzymes for therapeutic effect.

Selection of caging strategies for DNAzymes demands careful consideration. Caging strategies that separate into individual units may demonstrate improved stability and cellular uptake while in the caged formation, but the DNAzyme, once separated and in the absence of binding targets may be readily degraded by cellular enzymes, thus preventing accumulation to reach potential therapeutic thresholds. Degradation may also result in toxicity owing to the release of monomers or fragments with affinity for intracellular targets, such as RNA binding proteins.

## Carrier Molecule Modifications

Targeting ligands or carrier molecules are molecules that guide potential therapeutic molecules to a specific target cell or organ. In general, carrier molecules are either conjugated to or encapsulate the therapeutic molecule, and improve its efficacy through sustained delivery, enhanced transiting of biological barriers, specific cell, or organ targeting, and/or increased biocompatibility. Carrier molecules typically do not increase DNAzyme stability and thus are often used in conjunction with other chemical modifications, or as part of a larger nanoparticle structure.

Carrier molecules can be divided into several classes, including small molecules (biotin, vitamin B9 (folic acid) [105], carbohydrates and lipids (cholesterol, tocopherol) [93], etc.), peptides (eg, cell penetrating peptides, protein domains, antibodies (ie, monoclonal and polyclonal), aptamers [82,106], viral vectors [107], and other molecules such as 2′-*O*-hexadecyl [14,108] some of which have been applied to DNAzymes [109,110]. Each carrier molecule has its own advantages and disadvantages. Discussion of carrier molecules is beyond the scope of this review, a detailed review on these molecules is provided elsewhere [111,112].

## Conclusion

The uncommon properties of DNAzymes structure ensure highly specific gene modulation, in comparison with alternative antisense methods, including RNase H competent ASOs and siRNAs [18]. Owing to cleavage only at specific junctions, off-target annealing would not result in cleavage of nontargeted transcripts. DNAzyme technology has some additional advantages; low-cost DNA oligonucleotide synthesis, self-catalytic ability, and multiple turnover RNA cleavage [19] making DNAzymes very attractive candidates for molecular therapeutics, biosensing, and recently, DNA computation (programmable nanodevices based on DNAzymes) [113–115].

Despite the relatively low intracellular stability of DNAzymes and the requirement for cofactors, DNAzymes show great promise. This review highlights potential strategies to overcome some of the current limitations of DNAzymes as therapeutic agents. The translation of DNAzyme-mediated gene regulation to a clinical setting will continue to progress as novel modifications and strategies are developed and improved upon. Although delivery methods improve cellular uptake of nucleic acids, they do little to improve DNAzyme stability once released into the cytoplasm. Conversely, chemical modification of DNAzymes can increase stability, but generally do not improve cellular uptake.

Rational design and a better understanding of how a diversity of modifications interact and influence DNAzyme folding and catalytic action is essential for further development and optimization of DNAzymes for clinical application. Although the use of *in vitro* cleavage assays is a routine method to assess the cleavage kinetics of DNAzymes, this approach is limited because of lack of complexity in the reactions. Difficulty in transferring these cleavage rates to *in cell* and *in vivo* systems and predicting functionality highlights the need for testing within these systems at the forefront.

Evolution of oligonucleotides for eventual clinical intervention is ongoing. Novel chemistries developed initially for one mechanistic approach may be exploited to enhance the functionality of another. Although gapmers (RNase H-dependant ASOs) consisting of 5-10-5 designs with 2′-*O-*Me or MOE-modified bases in the 5′ and 3′ binding arms and PS linkages throughout the molecule are the industrial standard for gene knockdown, chemical modifications in the DNA core are limited to backbone modifications (PS linkages), with base modifications being incompatible with the gapmer mode of action, preventing RNase H activation. That the PS backbone can exert toxicity through nonantisense effects is widely reported [64,94,96,116–122], and may be among the reasons for the recent discontinuation of a number of RNase H ASO clinical studies. DNAzymes have an advantage in this regard, a DNAzyme may be more readily stabilized, and can tolerate or benefit from number of chemical modifications in the arm regions and at least some bases in the catalytic core.

Residues in the core that appear to be tolerant to a variety of modifications are G2, T8, and G14, whereas other nonconserved residues have been shown to tolerate at least some chemistry modifications; consistent with reports that certain nucleotides are essential for function, whereas others are nonessential (some are highly conserved, some are less conserved or redundant), being replaceable with alternative nucleotides, or even removable [75,123]. Some synergy might be derived from multiple modifications simultaneously, such as the combination G2 and T8 FANA chemistry substitution, not observed when the bases were modified individually. Further work is necessary to identify if this synergistic increase in cleavage activity is owing to a specific enhancement of DNAzyme folding and can be applied generally to other chemistries or owing to the unique characteristics of the FANA chemistry.

Modification of less conserved DNAzyme residues may be more effective than modifications to the highly conserved residues essential for function, such as T4, with its unique “out-of-frame flip” essential for folding into an active state B_1_ [23] or the aromatic base stacking action of residue G6 [23]. These essential functions should be taken into consideration when determining the best type and positioning of modified bases in the core of the DNAzyme.

Although an optimized DNAzyme, with maximized cleavage efficiency is the ideal from a chemistry perspective, from a biological and clinical perspective for application *in vitro* and eventual use of DNAzymes as therapeutic agents, selection of chemical modifications that are safe and are well tolerated is essential, thus limiting the selection of modifications to chemistries supported by data suggesting acceptable safety and tolerability *in vivo*.

A final consideration that is often overlooked for the translation of DNAzymes *in vivo* is the potential for DNAzymes to self-dimerize, forming inhibitory stem loop–like structures, which may partially account for the loss of DNAzyme catalytic ability when moving to an *in vivo* system. Because of the presence of the conserved 15 nucleotide catalytic core, DNAzymes are typically a minimum of 25–27 nucleotides long, but to a maximum of ∼37 nucleotides in length. Thus, the potential exists for self-dimerization of (1) the core to a binding arm or itself or (2) 5′ and 3′ binding arms to each other.

The concept of chemically modified oligonucleotide drugs that are innately stable and readily internalized by cells is attractive in drug development, and could be achieved by combinations of chemical modifications and conjugation or encapsulation into a nanoparticle. Ideally, such chemical modifications would be readily manufactured, biocompatible (nontoxic and nonimmunogenic), and maintain DNAzyme catalytic activity.
